# The fate of leaked heavy metals in the urban environment under different persistence and precipitation scenarios

**DOI:** 10.1038/s41598-024-59057-9

**Published:** 2024-04-09

**Authors:** Mehrdad Ghasemi, Touran Feyzi Kamareh, Maryam Morovati, Farogh Kazembeigi, Navid Alinejad, Hossein Moein, Ghasem Hassani

**Affiliations:** 1 Department of Environmental Engineering, Faculty of Engineering, University of Tehran, Tehran, Iran; 2 Faculty of Forest Science, Gorgan University of Agricultural Sciences and Natural Resources, Gorgan, Iran; 3 Department of Environmental Sciences and Engineering, Faculty of Agriculture and Natural Resources, Ardakan University, Ardakan, Iran; 4 Department of Environmental Health Engineering, School of Health, Ilam University of Medical Sciences, Ilam, Iran; 5 Department of Public Health, Fasa University of Medical Sciences, Fasa, Iran; 6 Department of Environmental Health, Health Promotion Research Center, Zahedan University of Medical Sciences, Zahedan, Iran; 7 Department of Environmental Health Engineering, School of Health, Yasuj University of Medical Sciences, Yasuj, Iran

**Keywords:** Hazardous waste, Waste management, Tobacco waste, Heavy metals, Environmental social sciences, Chemistry

## Abstract

The use of tobacco will lead to the littering of a large number of filters, and the leakage of pollutants from them into the urban environment is a serious concern. The aim of this study was to analyze the leakage of heavy metals from filter and estimate the annual concentration of pollution leakage in different waste routes and different climatic conditions. The results showed that the highest and lowest density of filter in the studied urban environment were 0.51 and 0.01 number/m^2^, respectively. According to the estimated annual production of 306 million cigarette butts in the studied area, the leakage of the studied metals was estimated to be 401 g. The share of copper, chromium, and cadmium from the total leakage was 67%, 8.3%, and 1.88%, respectively. The leakage of studied metals in rainy conditions was 2.86 times more than sunny conditions. In different scenarios, the minimum and maximum annual leakage of metals were estimated 23,043 and 350,419 mg/year, respectively. Filters are a little but important source of heavy metal emission into the urban environment, the amount of pollution from which is affected by the consumers’ behavior and the efficiency of the urban cleaning system. Education on the correct disposal of filters and increasing the efficiency of the urban cleaning system will lead to a reduction in pollution caused by tobacco consumption.

## Introduction

Population growth and urbanization have caused various environmental problems, including the production of significant volume of solid waste^1^. The consumption of more diverse products has led to the production of new solid waste types, some of which are the origin of serious pollutants such as pathogens and heavy metals^2^. The direct emission of pollution from solid waste and the indirect emission of pollutants from different stages of waste management, such as landfill leachate and incinerator exhaust, are important concerns^3^. For example, can mention the increase in the consumption of alkaline batteries in recent decades in various household appliances, which has caused the production of battery waste as a household hazardous waste^2^. Although the weight ratio of battery waste is very small, the leakage of heavy metals from them has been reported in significant amounts^2^. Therefore, paying attention to emerging solid wastes and the pollution caused by them is a necessity to protect the environment and reduce health risks.

Cigarette filters are one of the emerging wastes that have been produced since 70 years ago by the widespread consumption of filtered cigarettes in the world^4^. Increasing awareness of the dangers of cigarette smoke for smokers in the 1950s and efforts to reduce the concentration of smoke in the lungs led to the addition of filters to previous cigarettes^5^. The cigarette filter consists of Y-shaped fibers of cellulose acetate, which has the ability to trap cigarette smoke pollutants^6^. Although the cigarette filter can reduce the health consequences of smoking, but the production of cigarette filters as a solid waste caused by the smoking is inevitable^7^. Therefore, due to the function of the filter in trapping cigarette smoke pollutants, the cigarette filter contains various chemicals that make it known as a hazardous waste^8^. The important pollutants of cigarette filters include nicotine, cyanide, radioactive elements, PAHs, and heavy metals, that variety of which has been reported up to thousands of chemicals^1,9^. The origin of various pollutants, including heavy metals, can be the soil of tobacco cultivation. Also, the cigarette production process in the factory can increase the concentration of heavy metals in tobacco^10^. Therefore, different brands of cigarettes can contain different amounts of heavy metals, which is one of the important reasons for the variation of pollutants in the cigarettes filters^5^. However, the filter does not have the ability to keep the trapped metals for a long time, and these pollutants will gradually leak from cigarette filter^1,5^.

Although cigarette filters are a hazardous waste containing various pollutants, this is not the only relevant challenge. The large number of cigarette filters produced is one of the most important challenges in the management of this waste, as the annual production of 5 billion cigarette filters per year was reported^11^. However, it is predicted that the amount of production of this hazardous waste will increase to 8 billion equivalent to 2 million tons per year in less than 2 years^1,12^. Therefore, cigarette filters are an abundant waste and the source of various pollutants, including heavy metals. Another challenge in cigarette filter management is its disposal by smokers. The results of studies in different countries showed that most smokers littering cigarette filters, which makes it difficult and expensive to collect this waste^13^. These conditions have caused cigarette filters to be known as one of the most common litter in the urban environment and in public places such as beaches^14^. The lack of a regular cleaning system in public places such as beaches, as well as the inefficiency of the urban cleaning system in collecting littered cigarette filters, will cause them to persist^1,11,15^ which can lead to uncontrolled leakage of pollutants trapped in the filter. In addition, the current process of solid waste management, which in most cases ends up in landfills or incinerators, will cause the release of cigarette filter pollutants into the air and water sources^7,16^.

Studies on cigarette filters as a waste increased from 20 years ago. These studies can be classified into three parts, including cigarette filter dispersion in public environments, cigarette filter pollutants and their effects, and cigarette filter recycling. Cigarette filter pollutants were one of the important issues of past studies^5,11^. Cigarette filter pollutants are caused by its function in trapping cigarette smoke pollutants, so the composition of cigarette filter pollutants is expected to be similar to cigarette smoke pollutants^17^. Past studies investigated various pollutants including heavy metals^18^, PAHs^16^, toxins, and nicotine in cigarette filters^19–21^. Also, the impact of these pollutants on different organisms has been evaluated. In past studies, the effects of toxicity, growth changes, and mortality caused by exposure to cigarette filters on snails, fish, baby frogs, and even plants have been proven^22,23^. Also, in past studies, the amount of leakage of pollutants such as PAHs from the littered cigarette filter and the changes in the concentration of heavy metals in landfill leachate in different proportions of the cigarette filter have been investigated^16,20^. The aim of this study is to estimate the leakage of heavy metals from the littered cigarette filters. The aim of the leakage analysis in different scenarios was to consider the effect of climatic conditions and the efficiency of the urban cleaning system on it. Using the simultaneous estimation of heavy metal leakage from cigarette filter and considering scenarios based on climatic conditions and the efficiency of urban cleaning system is introduced as the innovation of this study. The results of this study and the estimation of the leakage of pollutants from cigarette filters in different scenarios can be used as a decision-making tool in urban management. This way of evaluating the environmental consequences of each of the waste management methods that are considered in the studied scenarios will help to choose the best option.

## Method

### Study area

This study was conducted in Yasuj, Iran. The per capita production of solid waste in this city was 640 g per day. The amount of filtered cigars consumption in the city was estimated based on the proportion of the city’s population compared to the consumption rate in the whole country. The studied area was categorized in three residential, recreational and commercial groups based on the difference of land-use. Based on this, four locations were selected from each land-use to study the density of littered cigarette filters, so a total of 12 locations were investigated by visual survey method^16^. In this method, based on a standard protocol, each location was studied in the hours before sunset^1,9^. The investigation of each location was repeated three times, in each observation, 20 cigarette filters were collected from each location, and after separating the environmental pollution, the weight of the sample was analyzed.

### Measurement of heavy metals

**Figure 1:**
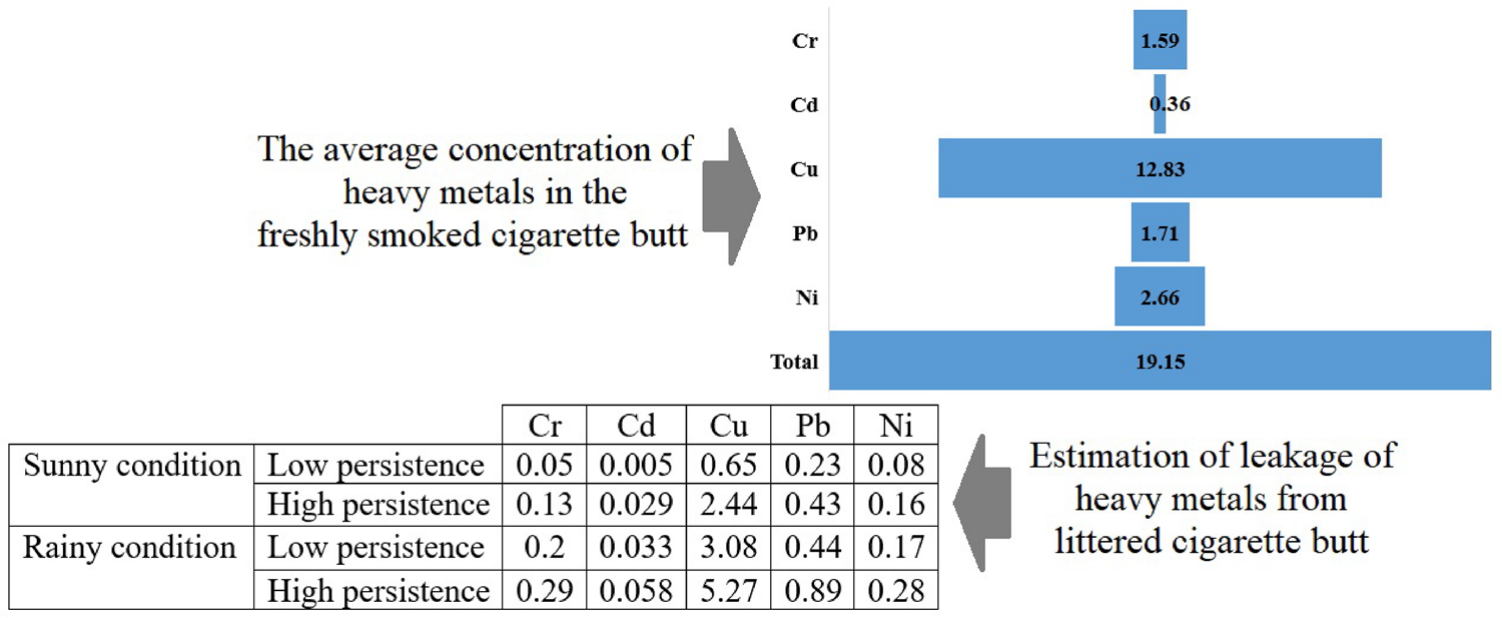
Concentration and leakage of heavy metals in cigarette butts (µg/g)^6,19^.

Estimation of heavy metal leakage was done based on indirect model and comparative analysis of concentration in freshly smoked cigarette filters and littered cigarette filters^19^. In this method, the concentration of the studied metals in cigarette filters caused by smoking popular brands is analyzed and then compared with the concentration of metals in samples collected from the urban environment. Based on this, the difference in the average concentration of metals in two categories of cigarette filters will indicate the leakage of metals from the cigarette filters into the environment^20^. The widely used method in the extraction of heavy metals includes the immersion in an acidic solution, and the estimation of leakage in different climatic conditions is done by simulating the intensity and duration of rainfall^21^. Based on this, the estimation of the studied heavy metal leakage is shown in Fig. 1.

### Scenarios

In this study, five scenarios were assumed for the leakage of heavy metals affected by the persist time of littered cigarette filters and precipitation conditions. It was reported that the persistence of cigarette filters in the urban environment is influenced by the efficiency of urban cleaning system and also the ratio of low access point^1^. Considering the effect of humidity on the intensity and concentration of leakage of cigarette filter pollutants, including heavy metals^24^, this effective factor was evaluated by the changes of precipitation in the studied area. In fact, changes in precipitation in different days of the year was considered as a factor in the studied scenarios due to its effect on the concentration of heavy metal leakage from the cigarette filter. According to the aims of this study, changes in the concentration of metals after leakage from the cigarette filter in the soil and even in the leachate landfill were not considered. These changes can be evaluated in future studies by soil and leachate analysis. Based on these variables, the conditions leading to the definition of scenarios are stated in Table 1. The first and second scenarios were the closest conditions to the current status, in which more than 90% of cigarette filter littering in the urban environment including 10% low access points was defined. The difference between these two scenarios was in the climatic conditions. In the third scenario, the variable conditions of the persistence of cigarette filters and the ratio of precipitation were defined. In the fourth and fifth scenarios, the behavior change of smokers and the reduction of littering were defined in order to estimate its effect on the reduction of heavy metal leakage.

**Table 1:** List of studied scenarios based on possible conditions.

Conditions	
C1	littering cigarette filters in the accessible environment
C2	littering cigarette filters in low access point
C3	Rainfall or irrigation in parks and lawns
C4	Dry environments and sunny days
C5	Proper disposal of cigarette filters in the trash bin
Scenarios	
S1	10% in C5, 80% in (C1.C4), 10% in (C2.C4)
S2	10% in C5, 80% in (C1.C3), 10% in (C2.C3)
S3	10% in C5, 70% in (C1.C4), 10% in (C1.C3), 10% in (C2.C3)
S4	50% in C5, 30% in (C1.C4), 10% in (C1.C3), 10% in (C2.C3)
S5	80% in C5, 10% in (C1.C4), 5% in (C1.C3), 5% in (C2.C3)

## Result and discussion

The results showed that the number of littered cigarette filters in the studied city had spatial and temporal variation. The density of littered cigarette filters in the worst sample was 0.51 number/m^2^. While the lowest observed littered cigarette filter was 0.01 number/m^2^. As the results of counting littered cigarette filters in Table 2 show, the average number of littered cigarette filters was 0.18 number/m^2^. The difference in the density of littered cigarette filters in the most polluted location and the cleanest location was 51 times. For example, in L3 and L6 and 0.51 number/m^2^ and 0.36 number/m^2^ littered cigarette filters were observed, respectively, which showed that they were the most polluted studied locations. While in L2 and L7 0.01 number/m^2^ littered cigarette filters were observed, respectively, which showed that they were the cleanest studied locations. There is a question in the difference in the density of littered cigarette filters in the studied locations. What factors cause the difference in the number of littered cigarette filters in different locations and at different times? The results of previous studies on littered waste, including littered cigarette filters, have shown that the reasons for the special variation and variation of urban litter include the difference in land-use and the difference in the efficiency of the urban cleaning system^1,9^.

**Table 2:** Density of littered cigarette filter in studied locations (number/m^2^).

	Locations											
	L1	L2	L3	L4	L5	L6	L7	L8	L9	L10	L11	L12
Density	0.12	0.01	0.51	0.18	0.08	0.36	0.01	0.24	0.27	0.04	0.15	0.19

The number of littered cigarette filters is directly related to the number of smokers and their behavior in disposing cigarette filters^25^. While most smokers littering cigarette filters^26^, in locations where the density of people is higher, due to the increase in the of smokers, the possibility of cigarette filters littering will be more likely^27^. These conditions cause the number of littered wastes, including cigarette filters, to be higher in commercial land-use where the density of citizens is higher than in other land-uses such as residential areas^28^. But in recreational land-use, in addition to the number of smokers, the location area is also effective in the density of litter, including littered cigarette filters. Although in other studies, the density of litter in recreational land-use is reported to be less than that of commercial land-use^1,29^, but the results of observations showed that in parts of a recreational land-use where people stop more, such as around park benches, the density of counted cigarette filters were increase. In addition, other factors effect on the density of littered cigarette filters. One of the effective factors is the efficiency of the urban cleaning system^14^. Considering that littering some waste, especially cigarette filters, is a common behavior among most citizens, the existence of a cleaning system is one of the necessities of urban management. However, the efficiency of the urban cleaning system may not be the same in all parts of the city, which can be one of the reasons for the spatial version of the littered cigarette filter^1,5^. Although the difference efficiency can be caused by the management structure and service quality, however, in the case of littered waste, especially cigarette filters, which are small in size, an important factor in the efficiency of the urban cleaning system is the number of low access points^30^. Tree pits, urban transport stations, and bicycle stations are the most important low access points that are more difficult to clean^28,30^. The existence of these points causes the accumulation of litter, including cigarette filters. Therefore, the increase in the number of low access points is directly related to the increase in the number of littered cigarette filters. Therefore, various factors that are effective in the number and persist of cigarette filters, affect the amount of heavy metal leakage from this hazardous waste.

The quantity of heavy metal leakage from cigarette filters depends on three main factors, including the initial concentration in the cigarette filters, the method of disposal of cigarette filters (persistence in the environment), and climatic conditions^19^. The estimation of the initial concentration of studied heavy metals in cigarette filters was 1.59–19.15 µg/gr. This concentration was consistent with the analysis of metal concentrations in popular cigarette brands in the country^19^. The concentration of different metals in the freshly smoked cigarette filters is not the same as that of littered cigarette filters^6^. The estimated initial concentration of lead in the cigarette filter was 1.71 µg/gr, while the concentration of cadmium and chromium was estimated at 0.36 µg/gr and 1.59 µg/gr, respectively^6,19^. Also, due to the leakage of pollutants from cigarette filters, the average concentration of metals in littered cigarette filters was estimated to be 5.3–35.4 percent lower than freshly smoked cigarette filters. The estimated leakage of metals from littered cigarette filters in low access points, which provide more persistence of cigarette filters, was 73% higher.

**Figure 2:**
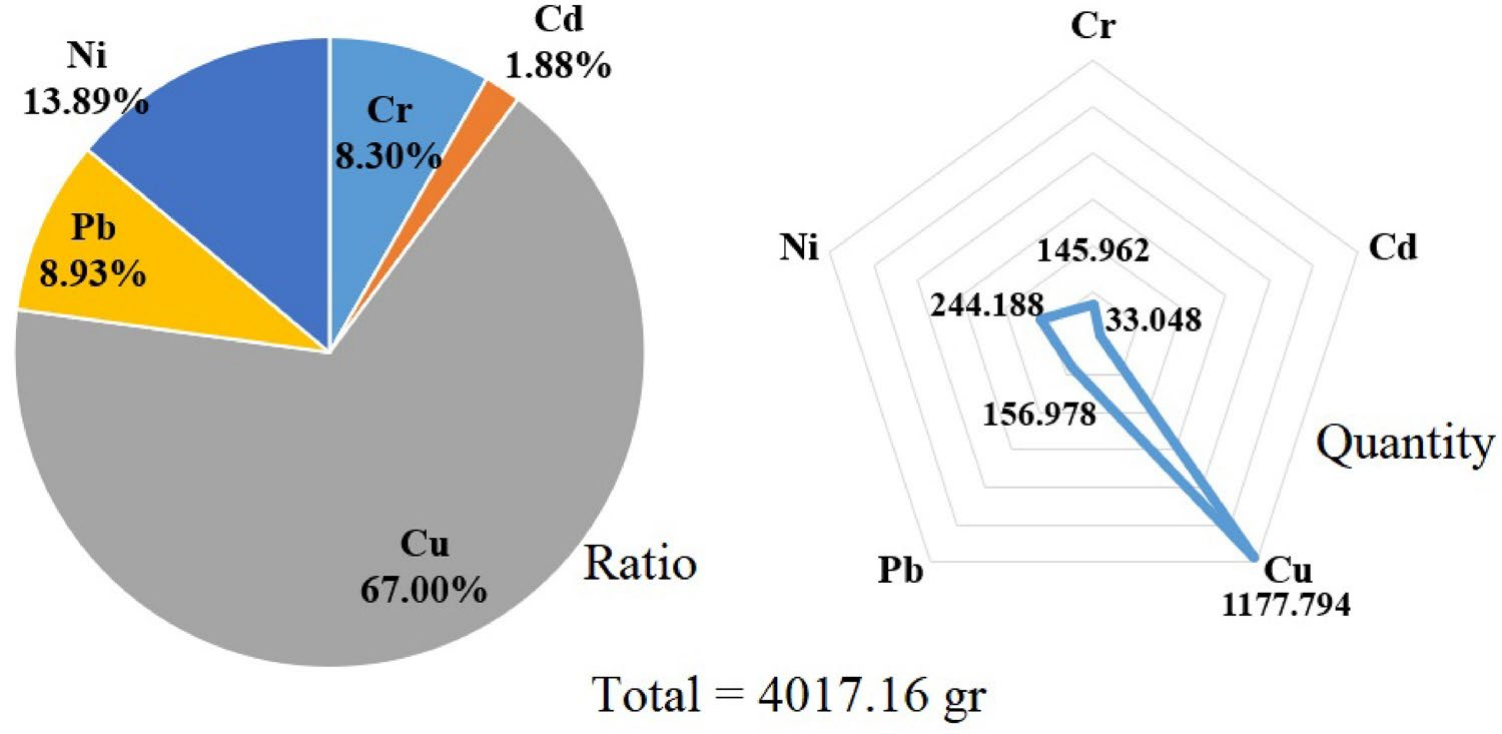
The annual leakage of heavy metals from cigarette butts in the study area.

As shown in Fig. 2, considering the smoking of 306 million filtered cigarettes, the annual leakage potential of the studied metals was estimated to be 4017 g. The highest concentration included cupper (1177 g) and the lowest concentration included cadmium (33 g). The reason for the difference in the concentration of metals in cigarette filters is the difference in the concentration of pollutants in cigarette filters affected by factors such as the difference in tobacco, the difference in smoking behavior, the difference in the cigarette production, and the difference in filter quality^31^. In fact, the origin of cigarette smoke pollutants that are finally trapped in the cigarette filter is the determining factor in the concentration of pollutants in the cigarette filter. For example, one of the origins of pollutants in tobacco is farm soil, which can have different concentrations of metals, and the use of different pesticides can cause the accumulation of different concentrations of this pollutant in tobacco leaves^30^. Also, the amount of smoke passed through the filter, which is directly dependent on the smoking behavior, and the concentration of the pollutant trapped in the filter, which is directly affected by the quality of the filter, is the reason for the difference in the concentration of the studied pollutant in the cigarette filter^23,32,33^.

**Figure 3:**
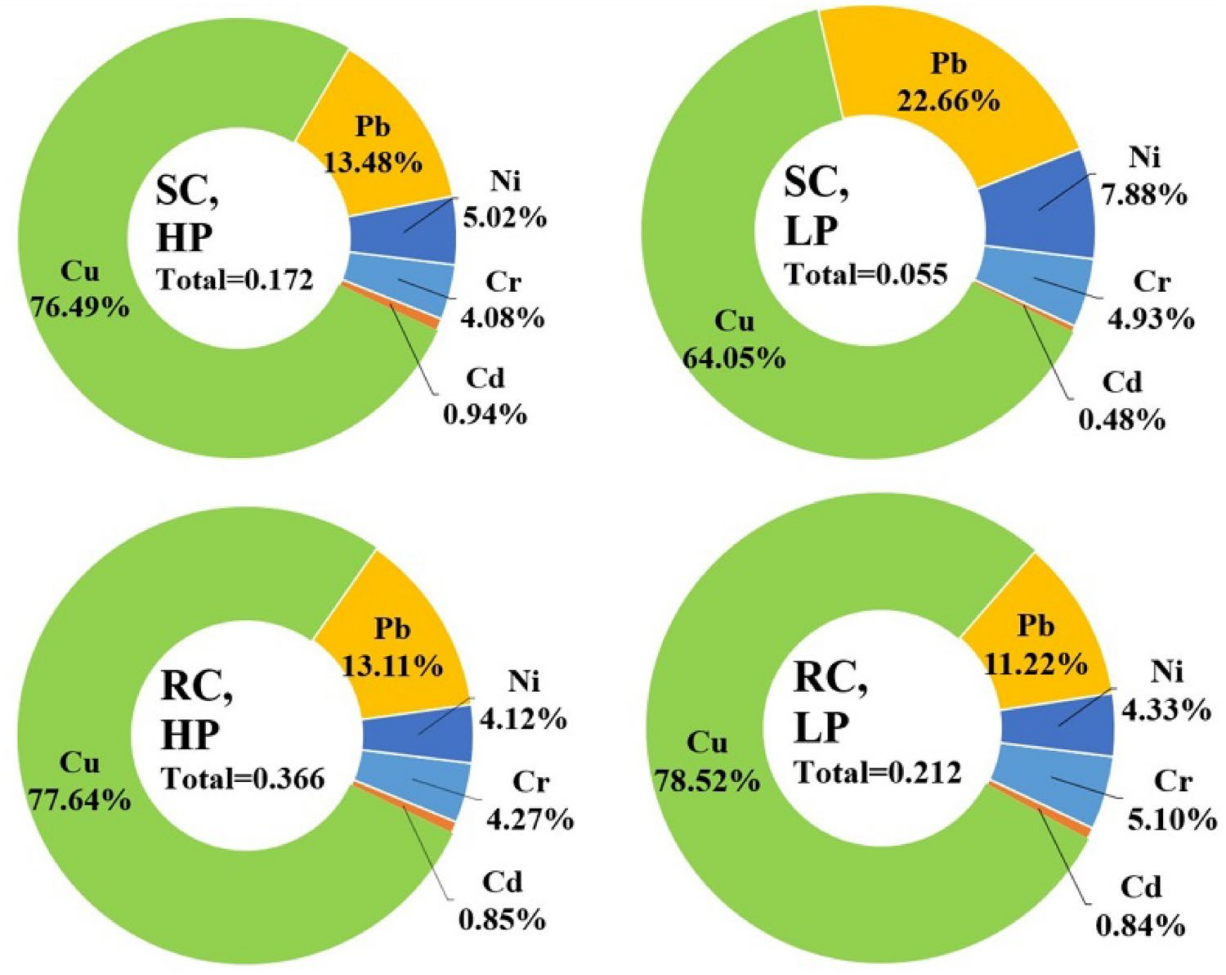
Concentration (µg/g) and ratio of metal leakage in the studied conditions.

**Figure 4:**
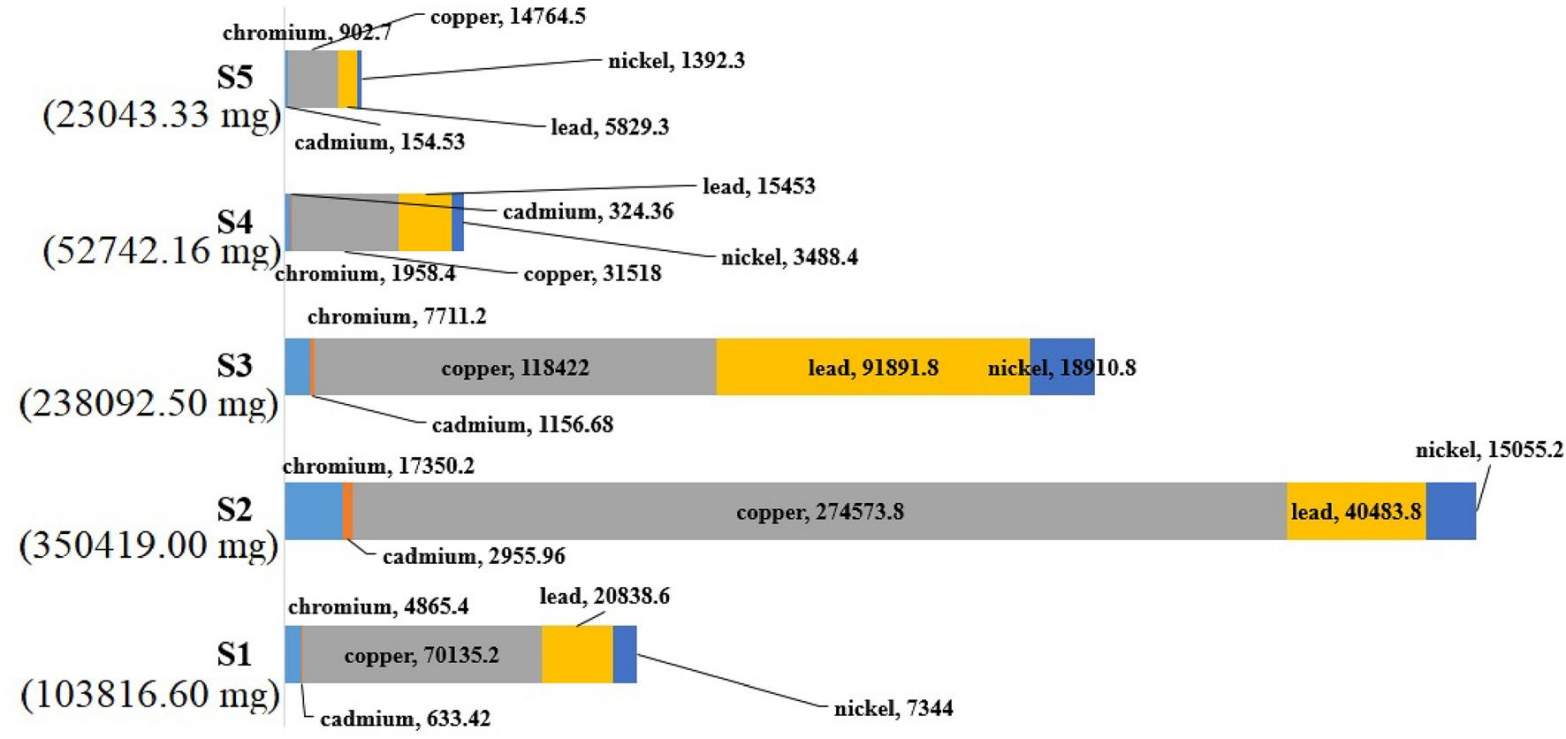
Annual leakage of metals from cigarette butts in the studied scenarios.

Table 3 shows the estimation of metal leakage considering rainfall and the persistence of littered cigarette filters in the environment. According to the density of cigarette filters in the studied locations, metal leakage was on average 0.201 µg/g. However, the average leakage of the studied metals was 3.86 times higher during the rainy condition, while the increase in the persistence increased the leakage of metals by 73.03%. Also, the impact of land-use on the density of littered cigarette filters caused the average leakage of metals in commercial areas to be estimated by 115.62% higher than in residential areas. The results showed that the leakage ratio of different metals was not the same. The highest ratio of leakage included lead (25.73%) and cupper (24%), respectively, while the nickel leakage ratio was the lowest value among the studied metals (6.33%). Therefore, in addition to the fact that the initial concentration of heavy metals in the cigarette filters is different, the ability of the filter to hold the trapped metals is not the same^10^. Based on this, the calculation of heavy metal leakage based on density variables, persistence in the environment, and climatic conditions in the studied scenarios is shown in Fig. 3. Also, as shown in Fig. 4, the results showed that the highest estimate of heavy metal leakage (350 g/year) was seen in S2, while the lowest leakage of the studied metals (23 g/year) was seen in S5.

**Table 3:** Metal leakage concentration in the studied conditions (µg/g).

	Locations											
	L1	L2	L3	L4	L5	L6	L7	L8	L9	L10	L11	L12
Sunny condition												
High persistence												
Cr	0.00468	0.00039	0.01989	0.00702	0.00312	0.01404	0.00039	0.00936	0.01053	0.00156	0.00585	0.00741
Cd	0.00108	0.00009	0.00459	0.00162	0.00072	0.00324	0.00009	0.00216	0.00243	0.00036	0.00135	0.00171
Cu	0.08784	0.00732	0.37332	0.13176	0.05856	0.26352	0.00732	0.17568	0.19764	0.02928	0.1098	0.13908
Pb	0.01548	0.00129	0.06579	0.02322	0.01032	0.04644	0.00129	0.03096	0.03483	0.00516	0.01935	0.02451
Ni	0.00576	0.00048	0.02448	0.00864	0.00384	0.01728	0.00048	0.01152	0.01296	0.00192	0.0072	0.00912
Low persistence												
Cr	0.0018	0.00015	0.00765	0.0027	0.0012	0.0054	0.00015	0.0036	0.00405	0.0006	0.00225	0.00285
Cd	0.0002	0.00002	0.0008	0.0003	0.0001	0.0005	0.00001	0.0003	0.0004	0.0001	0.0002	0.0002
Cu	0.0234	0.00195	0.09945	0.0351	0.0156	0.0702	0.00195	0.0468	0.05265	0.0078	0.02925	0.03705
Pb	0.00828	0.00069	0.03519	0.01242	0.00552	0.02484	0.00069	0.01656	0.01863	0.00276	0.01035	0.01311
Ni	0.00288	0.00024	0.01224	0.00432	0.00192	0.00864	0.00024	0.00576	0.00648	0.00096	0.0036	0.00456
Rainy condition												
High persistence												
Cr	0.01044	0.00087	0.04437	0.01566	0.00696	0.03132	0.00087	0.02088	0.02349	0.00348	0.01305	0.01653
Cd	0.0021	0.00017	0.00887	0.00313	0.00139	0.00626	0.00017	0.00417	0.00469	0.00069	0.0026	0.00330
Cu	0.18972	0.01581	0.80631	0.28458	0.12648	0.56916	0.01581	0.37944	0.42687	0.06324	0.23715	0.30039
Pb	0.03204	0.00267	0.13617	0.04806	0.02136	0.09612	0.00267	0.06408	0.07209	0.01068	0.04005	0.05073
Ni	0.01008	0.00084	0.04284	0.01512	0.00672	0.03024	0.00084	0.02016	0.02268	0.00336	0.0126	0.01596
Low persistence												
Cr	0.0072	0.0006	0.0306	0.0108	0.0048	0.0216	0.0006	0.0144	0.0162	0.0024	0.009	0.0114
Cd	0.0011	0.0001	0.00504	0.00178	0.00079	0.00356	0.0001	0.00237	0.00267	0.00039	0.00148	0.00188
Cu	0.11088	0.00924	0.47124	0.16632	0.07392	0.33264	0.00924	0.22176	0.24948	0.03696	0.1386	0.17556
Pb	0.01584	0.00132	0.06732	0.02376	0.01056	0.04752	0.00132	0.03168	0.03564	0.00528	0.0198	0.02508
Ni	0.00612	0.00051	0.02601	0.00918	0.00408	0.01836	0.00051	0.01224	0.01377	0.00204	0.00765	0.00969

S2 and S3 show the effect of rainfall on the leakage of heavy metals, and S4 and S5 show the effect of changing the behavior of smokers on the leakage of heavy metals. Humidity will increase the leakage of pollutants from the cigarette filters^24^, which was 386% for the studied heavy metals. This ratio will be higher in low access points due to the longer persistence of cigarette filters in the urban environment, which was seen in the estimated leakage in S3 and S2. In addition, changing the behavior of smokers and reducing littering had a significant impact on preventing pollution in the urban environment. The 77.6% reduction in estimated leakage in S5 compared to S1 showed that the education of smokers and changing their behavior is very important. The leakage of 350 g of heavy metals per year into the urban environment is known as a serious threat to the environment and the health of citizens. Cigarette filter is a hazardous that its trapped pollutants, including heavy metals, can have adverse effects on citizens’ health by entering the food chain^34^. Also, leakage of heavy metals into groundwater resources is one of the consequences of littering cigarette filters by smokers^21^.

Considering the impact of cigarette filter persistence in the urban environment on the increase of heavy metal leakage, the efficiency of the urban cleaning system is an important factor in reducing the negative consequences of littered cigarette filters. To increase the efficiency of cigarette filter management, increasing the number of waste containers or designing special bins for cigarette filters can be proposed as a solution. But the results of some studies have shown that even in places where there are enough waste containers in short distances, density of littered cigarette filters is significant^21^. Therefore, it is necessary to find a solution to increase the efficiency of the urban cleaning system in collecting littered cigarette filters and changing the behavior of smokers in the method of disposing of this hazardous waste.

The results of this study showed that cigarette filters are a waste with the potential to leak various pollutants, including heavy metals. These pollutants will cause many effects such as toxicity and genetic change in urban organisms^35–37^. The toxic effects of cigarette filters and its pollutants on snails and other studied organisms was confirmed^35^. Even the effect of this hazardous waste on the growth of plants has been proven^38^. Therefore, one of the main concerns of the annual leakage of 350 g of heavy metals from littered cigarette filters are the impact on urban organisms. Also, the entry of this hazardous waste and the leakage of its pollutants into the nests of birds and urban animals can be one of the environmental consequences of cigarette filters, because it has been reported that birds use cigarette filters to build nests^39^. In addition, the ingestion of cigarette filters by infants and pets is one of the concerns of littered cigarette filters, so that dozens of cases of cigarette filter ingestion have been reported annually in Italy, Japan, and the United States^22^. Although this study achieved its objectives, the following points are suggested for future studies.

- Evaluation of other trapped pollutants in cigarette filter such as PAHs and nicotine in scenarios similar to this study.
- Studying the consequences of estimated leaked pollutants on soil and water quality.
- Studying the impact of changes in the consumption of filtered cigarettes in the society over several years on the environment by changes in the leakage of pollutants.

## Conclusion

The density of littered cigarette filters in the urban environment and the leakage of heavy metals from them were investigated in different scenarios with persistence and humidity variables. The results showed that the density of littered cigarette filters varied due to the land-use and was on average 0.18 number per square meter. The leakage of studied heavy metals including lead, chromium, cadmium, cupper, and nickel from littered cigarette filters was estimated to be 238 g per year based on the average weight of 19.15 µg per gram of cigarette filters. The increase in the persistence of cigarette filters in the urban environment caused an increase in leakage up to 77.6%, and in rainy conditions, the increase in leakage of heavy metals was estimated up to 386%. The results showed that cigarette filters are one of the important sources of heavy metal release into the urban environment, which has negative consequences for the health of citizens and urban organisms, even plants. The results of this study can be used as a decision-making tool. This method helps to choose the best option with the least environmental and health consequences for managing cigarette filters as a hazardous waste. The estimation of the reduction of heavy metal leakage by change in the behavior of smokers and reducing the littering of cigarette filters was up to 93.4%. So, focusing on increasing the efficiency of the urban cleaning system and change in the smokers’ behavior is a necessity in urban pollution management.

## Data Availability

The datasets generated and analyzed during the current study were available from the corresponding author on reasonable request.
